# Evaluating Indoor Air Phthalates and Volatile Organic Compounds in Nail Salons in the Greater New York City Area: A Pilot Study

**DOI:** 10.3390/ijerph191912411

**Published:** 2022-09-29

**Authors:** Inkyu Han, Jin Young Seo, Dana Boyd Barr, Parinya Panuwet, Volha Yakimavets, Priya Esilda D’Souza, Heyreoun An-Han, Masoud Afshar, Ying-Yu Chao

**Affiliations:** 1 Department of Epidemiology and Biostatistics, Temple University College of Public Health, Philadelphia, PA 19122, USA; 2 Hunter College School of Nursing, The City University of New York, New York, NY 10010, USA; 3 Gangarosa Department of Environmental Health, Rollins School of Public Health, Emory University, Atlanta, GA 30322, USA; 4 Gulf Coast Center for Precision Environmental Medicine, Baylor College of Medicine, Houston, TX 77030, USA; 5 Department of Epidemiology, Human Genetics, and Environmental Science, University of Texas Health Science Center at Houston, Houston, TX 77030, USA; 6 School of Nursing, Rutgers, The State University of New Jersey, Newark, NJ 07102, USA

**Keywords:** phthalates, volatile organic compounds, community organizations, nail salons

## Abstract

The Greater New York City area ranks highest in the United States in the number of nail salon technicians, primarily Asian immigrant women. Nail salon technicians are exposed to toxic phthalates and volatile organic compounds daily in nail salons. The purpose of this pilot study was to measure a mixture of phthalates and volatile organic compounds in nail salons in the Greater New York City area, and to characterize work-related determinants of indoor air quality in these nail salons. Working with four Asian nail salon organizations in the Greater New York City area, we measured indoor air phthalates and volatile organic compounds at 20 nail salons from February to May 2021 using silicone wristbands and passive samplers, respectively. Nail salon characteristics were also examined. We measured six phthalates and 31 volatile organic compounds. Di(2-ethylhexyl) phthalate and Diethyl phthalate had the highest concentrations among the six phthalates measured. Concentrations of toluene, *d*-limonene, methyl methacrylate, and ethyl methacrylate were higher than that of the rest. Manicure/pedicure tables, the number of customers per day, and application of artificial nail (acrylic) services were positively associated with the levels of phthalates and volatile organic compounds. Given the large number of people employed in the nail industry and the even larger number of customers visiting such establishments, exposures to these toxic chemicals are likely to be widespread.

## 1. Introduction

The nail salon industry in the United States (U.S.) employed over 400,000 persons in 2018. Employment in the industry is projected to grow by 33 percent between 2020 and 2030, 4-fold faster than the U.S. average (8 percent) for all occupations [1]. The Greater New York City (GNYC) area ranks highest in the U.S. with respect to the number of nail salon technicians (NSTs) [1,2,3]. NSTs in the GNYC area are primarily Asian immigrant women of reproductive age [2,3,4]. Although Korean and Chinese immigrants constitute the primary NST workforce (estimated 52%) in New York State [5,6,7], to our knowledge, only one study has conducted exposure assessments among them [8].

Survey studies showed that NSTs are exposed to nail products on a daily basis, and the frequent use of nail products among NSTs has been linked to skin irritation, allergic reactions, and respiratory and reproductive symptoms [4,9,10,11]. Occupational health studies have examined the chemical ingredients of nail products and the risks of potential exposure to organic chemical compounds in nail salons. NSTs are exposed to toxic volatile organic compounds (VOCs) such as formaldehyde, benzene, toluene, and acrylates [12,13,14,15,16]. For example, formaldehyde is classified as a human carcinogen by the International Agency for Research on Cancer (IARC) and exposure to it is associated with irritation of the eyes, nose, throat, and skin [17,18,19,20]. Acrylates such as methyl methacrylate (MMA) and ethyl methacrylate (EMA) are known as skin irritants and can cause irritation of the upper respiratory tract [21,22].

NSTs are also frequently exposed to phthalates since NSTs use plasticizers while they provide nail service. Phthalates are endocrine disrupting chemicals that may be related to adverse reproductive effects [23,24,25]. Compared to VOC exposure studies, only a few studies examined phthalate exposures for NSTs [26,27]. The passive sampling method using a silicone wristband (SWB) has drawn attention for assessing exposure to semi-volatile organic compounds (SVOCs) including phthalates since it does not require active sampling pumps. Although SWBs have been recently used for the characterization of a mixture of SVOCs in various environmental and occupational settings [28,29,30,31,32], exposure to phthalates among NSTs is not fully explored.

This pilot study aimed to assess the feasibility of SWBs collecting airborne phthalates, measure the mixture of VOCs and phthalates at nail salons in GNYC, and characterize airborne exposure to phthalates and VOCs at nail salons between New Jersey (NJ) and New York (NY).

## 2. Materials and Methods

### 2.1. Recruitment of Nail Salons

Through our previous studies that examined the factors influencing health service utilization among Korean and Chinese immigrant NSTs [3,5], we successfully established community–academic partnerships with three Asian nail salon organizations and one non-profit community organization for the Korean and Chinese immigrant population in GNYC. As part of an ongoing community–academic research partnership, we conducted indoor air quality exposure assessments at 20 nail salons. We distributed a study advertisement via nail salon partners, Asian community organizations, and Korean and Chinese community websites. We received contact information of nail salon owners from nail salon partners and community organizations who indicated their interest in participating in our study. We also recruited nail salon owners who directly contacted the investigator via online advertisements. Nail salon owners were contacted over the phone, and the purpose of this study and data collection procedures were conveyed to them. If they agreed to participate in our study, an investigator scheduled an air monitoring campaign at their preferred time.

An investigator visited each nail salon twice, by appointment, approximately 72 h apart. During the first visit, we provided written information about this study and prior to deployment received verbal consent for voluntary participation. After completion of the consent process, we collected information about the nail salon environment with assistance from salon owners or managers. No personal or nail salon identification information were documented. Three days later (approximately 72 h from the deployment of air samplers), a field investigator revisited each nail salon to collect the air sampling badges. Each salon owner received USD 50 for their participation. Upon completion of this study, each nail salon received an individual report of their results by mail. All study materials, including recruitment, consent, field log questions, and participant reports, were provided in their preferred language (Korean, Chinese, or English). The study design and protocols were reviewed and exempted by the Institutional Review Board of Hunter College, the City University of New York (CUNY), as no personal or nail salon identification information was collected for this study.

### 2.2. Sample Collection

Two air sampling campaigns were conducted at 20 nail salons. The first sampling campaign was conducted at 10 nail salons between 25 February 2021 and 10 March 2021 and the second at an additional 10 nail salons between 31 March 2021 and 8 May 2021 During the first campaign, we deployed two passive organic vapor monitors (OVM) 3500 (3M Company, Saint Paul, MN, USA) per nail salon. During the second campaign, we deployed two 3M OVM badges for VOCs and a silicone wrist band (SWB) for phthalates per nail salon. Both OVM badges and SWBs were hung over or near a manicure/pedicure table (attached to the wires of the sneeze guard installed at the table for the prevention of potential exposure to SARS-CoV-2) for approximately 72 h to collect VOCs and phthalates, respectively. A total of 40 OVM badges were deployed at 20 nail salons and 10 SWBs were deployed at 10 nail salons during the entire sampling campaign. We calculated the average of VOCs from the two collocated OVM badges at each nail salon. The field investigator also recorded the start date and time and collected the work characteristics of the nail salons, such as work hours, the number of NSTs, the number of customers, type of ventilation, and the nail services provided. Three days after deployment of the samplers, the investigator revisited the nail salons to collect samples, and all collected samples were transferred to Hunter College and stored in a refrigerator. Stored OVM badges were shipped at 4 °C to the UTHealth exposure assessment laboratory and SWBs were shipped to Emory University. All collected samples were analyzed within one month of the completion of the sample collection. We sent the individual study results to all nail salons with additional information on the occupational exposure limit values from the Occupational Safety and Health Administration, recommended threshold limit values from the American Council of Governmental Industrial Hygienists, and typical environmental concentrations if the range of chemical concentrations was available.

### 2.3. VOC Analysis

A suite of 31 VOCs extracted from the OVM samplers was analyzed by an Agilent 6890 gas chromatograph coupled with a 5973 mass spectrometer (Agilent Technologies, Palo Alto, CA, USA). Detailed sample preparation and analytical conditions, including quality assurance and quality control (QAQC) have been described in previous studies [33,34]. We observed that biases of paired samples were less than 25 percent for all measured VOCs. We also deployed four field blank badges (20% of field samples) to determine potential contamination during sampling, transport, and analysis. Individual VOCs from blank badges were not detected or less than the limit of detections (LODs); thus, we did not observe substantial contamination during our experiment. Individual VOCs detected in blank badges were subtracted from corresponding VOCs measured from sampling badges. The airborne concentrations of 31 VOCs were determined based on the mass detected on OVM badges divided by the estimated sampling volume using the combination of Fick’s law and sampling time [33,34]. The unit of 31 VOCs is reported as ppb (*v*/*v*).

### 2.4. Phthalate Analysis

A suite of phthalates extracted from the SWBs was analyzed by the Laboratory of Exposure Assessment and Development for Environmental Research (LEADER) at Emory University. LEADER cleaned the SWBs prior to their deployment in the field using the following procedure. SWBs were rinsed with Milli-Q water to remove debris then soaked in a 1:1 ethyl acetate: hexanes (1:1 *v*/*v*) solution for two hours and a 1:1 ethyl acetate: methanol (1:1 *v*/*v*) solution for two hours to remove any residual contaminants. Next, they were vacuum oven dried at 100 °C for two hours before being stored in zip seal bags and shipped to CUNY for deployment. After deployment, the samples were shipped back to LEADER for phthalates analysis.

To extract the target compounds, ⅓ of each wristband was submerged in 10 mL hexane: dichloromethane (1:1 *v*/*v*), spiked with labeled internal standards, and sonicated for 10 min. The organic solution was loaded onto pre-conditioned Strata FL-PR Florisil 500 mg/6 mL solid phase extraction columns (Phenomenex, Torrance, CA, USA) and eluted with 10 mL ethyl acetate. This procedure was performed twice. Eluates were concentrated to dryness and reconstituted in 1 mL toluene and further diluted 20 fold with toluene before injection. Analysis of phthalate compounds was performed using a gas chromatograph (GC) coupled with a mass spectrometer (Agilent Technologies) in single ion monitoring mode. Seven calibrants, two quality control materials (50.0 ng/mL and 500 ng/mL), and blanks were analyzed concurrently with unknown samples. Quantification of phthalates was conducted using isotope dilution calibration. The calibrants were prepared using a mixture of nonane: toluene (1:1 *v*/*v*) to cover a quantification range of 2.00–5000 ng/mL. Concentrations of phthalates in each sample were background subtracted. The limit of quantification for six measured phthalates (Di(2-Ethylhexyl) phthalate (DEHP); Di-n-octyl phthalate (DnOP); Diisobutyl phthalate (DiBP); Diethyl phthalate (DEP); Di-n-butyl phthalate (DnBP); Benzylbutyl phthalate (BzBP)) was 2 ng per sample.

### 2.5. Data Analysis

The survey database was built by entering the information from the field log sheet and nail salon characteristics datasheet. After validation of the speciated VOCs and phthalates, all validated chemical data were merged into the survey database. For statistical data analyses, data from field log questions were summarized and described using proportions for categorical variables, and means and standard deviations (SD) were calculated for continuous variables. Differences in nail salon characteristics between NJ and NY were tested using t-tests and χ^2^ analyses at a significance level of 0.05. The data distribution of measured VOCs and phthalates was examined for the mean, SD, and selected percentiles, including the median. Because most chemical constituents were not normally distributed, we conducted Wilcoxon–Mann–Whitney tests to compare the median values of VOCs and phthalates between NJ and NY. We also compared the differences in medians for VOCs and phthalates by salon characteristics using the Wilcoxon–Mann–Whitney test. All data analyses were conducted using IBM SPSS Statistics for Windows, version 26 (IBM Corp., Armonk, NY, USA). A significance level of 0.05 was used for all analyses.

## 3. Results

### 3.1. Salon Characteristics

Table 1 summarizes the nail salon characteristics from 10 salons in Northeastern NJ and 10 salons in New York City and Long Island in NY. The characteristics of the nail salons varied: the mean business hours were 9.6 (range: 8.5–12.0), the mean number of employees was 7.6 (range: 2–17), and the mean daily number of customers was 36.3 (range: 14–85). Each salon had at least eight tables (four for manicures and four for pedicures). The mean of the total number of tables was 14.4 (range: 8–24).

Seventy percent of the nail salons (n = 14) had at least two doors and six had a front door only. While 14 of the salons did not have any windows, six had at least one window. Approximately two-thirds of the nail salons (n = 13) had a ventilation system (e.g., local exhaust ventilation (LEV), ceiling exhaust fan) and sixty percent of nail salons (n = 12) used some “organic products” with all-natural and organic ingredients. While most nail salons provide regular manicures and pedicures, ultraviolet (UV) gels, and dip powder nails services, only six provided acrylic nail services. In addition, some nail salons provided other beauty services, including spa/facials/waxing (n = 14), massage (n = 13), and eyelash extension (n = 7). There were no statistical differences in nail salon characteristics between the NJ and NY groups.

### 3.2. Descriptive Statistics of VOCs and Phthalates

**Figure 1:**
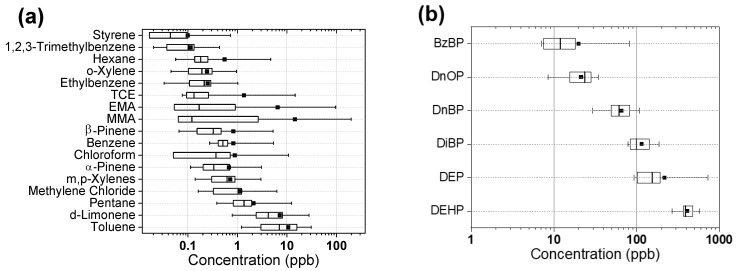
Boxplots of 18 VOCs and 6 phthalates measured in 20 nail salons. (**a**) shows a boxplot of 18 VOCs detected from at least 13 out of 20 nail salons. Tetrachloroethylene (TCE); ethyl methacrylate (EMA); and methyl methacrylate (MMA). (**b**) shows a boxplot of six phthalates detected from all 10 nail salons. Phthalates are Di(2-Ethylhexyl) Phthalate (DEHP); Di-n-octyl phthalate (DnOP); Diisobutyl phthalate (DiBP); Diethyl phthalate (DEP); Di-n-butyl phthalate (DnBP); Benzylbutyl phthalate (BzBP). Mean and median values are shown as squares and straight lines. Left and right edge of the boxes represent 25th and 75th percentiles. Whiskers represent minimum and maximum values.

Table 2 summarizes the mean and median concentrations of detected VOCs and phthalates. Pentane, methylene chloride, benzene, toluene, ethylbenzene, xylenes (BTEX), tetrachloroethylene, *α*-pinene, and *d*-limonene were detected at all nail salons. Additionally, β-pinene and 1,2,3-trimethyl benzene were detected at 19 nail salons, and hexane was detected at 18 nail salons. MMA was detected at seven nail salons in NJ and at three nail salons in NY, whereas EMA was detected at seven nail salons in NJ and six nail salons in NY. Of the 31 detected VOCs, median values of toluene (7.03 ppb) and *d*-limonene (4.21 ppb) were higher than that of the rest (Figure 1a). Among phthalates, six phthalates (DEHP, DnOP, DibP, DeP, DnBP, and BzBP) were detected in all 10 nail salons in this study (Table 2). We found that concentrations of DEHP (median = 391.80 ng/g-SWB) were the highest followed by DEP, DiBP, DnBP, DnOP, and BzBP (Figure 1b).

**Figure 2:**
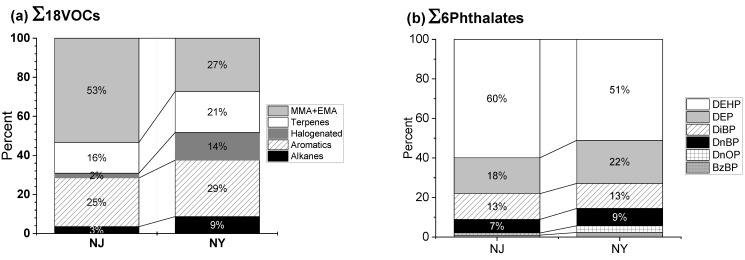
Contribution of categorized sum of 18 VOCs and 6 phthalates in nail salons. (**a**) shows the contribution of five VOC groups to the sum of 18 VOCs: (1) sum of MMA and EMA; (2) terpenes as the sum of α-pinene, β-pinene, and d-limonene; (3) halogenated as the sum of methylene chloride, chloroform, and tetrachloroethylene (TCE); (4) aromatics as the sum of BTEX, styrene, and 1,2,3-trimethylbenzne; and (5) alkanes as the sum of pentane and hexane. (**b**) shows the contribution of individual phthalates the sum of six phthalates. The percentage of both DnOP and BzBP at NJ and NY nail salons were less than 3 percent. Phthalates are Di(2-Ethylhexyl) Phthalate (DEHP); Di-n-octyl phthalate (DnOP); Diisobutyl phthalate (DiBP); Diethyl phthalate (DEP); Di-n-butyl phthalate (DnBP); Benzylbutyl phthalate (BzBP).

Figure 2a shows the relative contribution of each group of VOCs to the sum of the 18 VOCs in NJ and NY. The sum of the 18 VOCs was calculated using individual VOCs detected in at least 10 nail salons. Overall, the median of the sum of 18 VOCs was higher in NJ (59.25 ppb) than in NY (37.51 ppb). The sum of the 18 VOCs was attributed to MMA + EMA (53%), aromatic hydrocarbons (25%), and terpenes (16%) in NJ nail salons, and to aromatic hydrocarbons (29%), MMA + EMA (27%), terpenes (21%), halogenated VOCs (14%), and alkanes (9%) in NY nail salons. The relative contributions of MMA + EMA to the sum of 18 VOCs were greater in NJ (53 %) than in NY (27 %). Unlike VOCs, there were no differences in the relative contribution of phthalates to the sum of the six phthalates in nail salons between NJ and NY. The contributions of DEHP, DEP, and DiBP to the sum of the six phthalates were 91 percent and 86 percent at nail salons in NJ and NY, respectively (Figure 2b).

### 3.3. VOCs and Phthalates by Salon Characteristics

**Figure 3:**
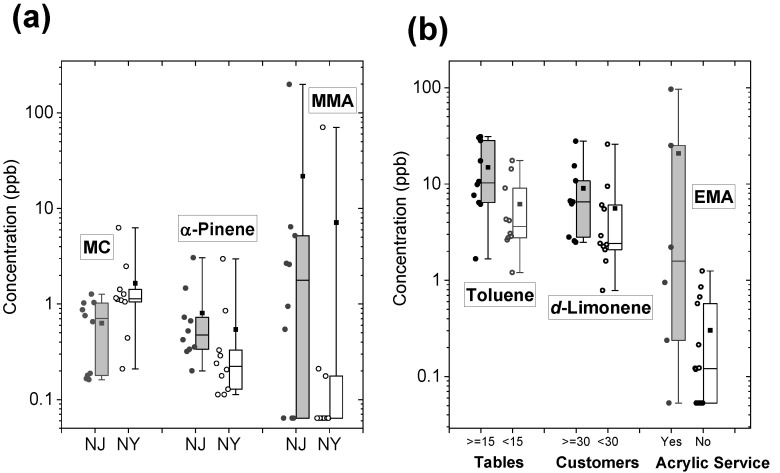
Box plots for comparison of VOCs and phthalates by sampling locations and selected nail salon characteristics. (**a**) shows the comparison of methylene chloride (MC), α-pinene, and meth methyl methacrylate (MMA). (**b**) compares the differences of toluene by the number of manicure and pedicure tables, d-limonene by average daily customers, and the comparison of ethyl methacrylate (EMA) by acrylic nail service.

Of the 31 VOCs and 6 phthalates, methylene chloride (*p* = 0.013) and DnOP (*p* = 0.017) were significantly higher at nail salons in NY than in NJ. The median concentrations of α-pinene (*p* = 0.034) and MMA (*p* = 0.048) in NJ nail salons were significantly higher than in NY nail salons (Figure 3a). Figure 3b shows that the median toluene concentration was significantly higher at nail salons with 15 or more manicure/pedicure tables than at nail salons with less than 15 tables (*p* = 0.042). In addition, nail salons serving more than 30 customers per day had significantly higher levels of d-limonene than nail salons serving less than 30 customers per day (*p* = 0.044, Figure 3b). Nail salons providing acrylic nail services showed significantly higher concentrations of EMA than nail salons without acrylic nail services (*p* = 0.049, Figure 3b).

## 4. Discussion

Working with four community groups, we conducted indoor air quality monitoring at 20 nail salons for about three months. This study was exclusively conducted at Korean and Chinese nail salons in NJ and NY, whereas previous studies focused primarily on nail salons employing or owned by Vietnamese individuals. Thirteen VOCs were frequently detected in at least 18 of the 20 nail salons and six phthalates were detected in all 10 nail salons. Generally, the median and mean concentrations of benzene and toluene were similar or lower than those reported in previous studies. This discrepancy might be related to the use of different nail products at nail salons located in other states or by different ethnic groups [35,36]. Another possible explanation is the different sample collection periods. We collected airborne VOCs for approximately 72 h, including non-business hours (e.g., from 8 pm to 10 am for three consecutive days), whereas existing studies collected samples during work hours only, typically 8 to 10 h. Thus, the levels of benzene, toluene, and other VOCs collected in our study, including non-business hours, are expected to be lower than those in previous studies.

Of the 20 nail salons, MMA and EMA were detected at 10 and 13 nail salons, respectively. The mean for MMA in this study was 14.44 ± 46.13 ppb. We found the concentrations of MMA and EMA are different between NJ and NY nail salons. The results may be associated with different policies between two states. The New York State General Business Law has prohibited the sale, use, and application of MMA by businesses, including nail salons, since 2014 [37]; therefore, as a substitute for MMA, NY nail salons are likely to use EMA for nail services. Although we did not examine the ingredients of all nail products in this pilot study, our results suggest that NY nail salons mainly use EMA (median: 0.55 ppb) instead of MMA (median: < 0.09 ppb). Hence, the higher levels of EMA in NY nail salons may be associated with the New York State Public Health Regulations. Conversely, NJ nail salons had lower concentrations of EMA (median: 0.12 ppb) than MMA (median: 1.77 ppb), suggesting that NJ nail salons in our study mostly used MMA for their nail services.

While previous exposure assessment studies focused on the measurement of BTEX, MMA, and EMA, other VOCs have not been comprehensively investigated for indoor air quality in nail salons. With our multiple VOC-targeted analysis approach, we observed that *d*-limonene (median: 4.21 ppb, mean ± SD: 7.14 ± 7.68 ppb) was the VOC with the second highest concentration following toluene. To our knowledge, other nail salon studies, except the Boston [38] and Michigan nail salon studies [15], have not measured or reported concentrations of *d*-limonene in nail salons. Although the measured *d*-limonene concentration in this study included non-work hours, the mean *d*-limonene was approximately 3- to 4-fold greater than that of Boston [38] and Michigan nail salons [15]. The elevated levels of *d*-limonene in all 20 nail salons in this study may be related to the use of cleaning and disinfection agents along with other fragrances [39,40] since *d*-limonene is not an active ingredient of nail products. Ceballos et al. (2019) also suggested that *d*-limonene in nail salons may be associated with the use of cleaning products rather than nail products [38]. Although we did not collect information about the use of cleaning and disinfection agents in this pilot study, the elevated levels of *d*-limonene in this study might be associated with the increased use of cleaning and disinfection products during the COVID-19 pandemic. On the day of deploying the samplers, we observed that NSTs cleaned the tables and chairs after providing nail service to each customer. Moreover, we found that the levels of *d*-limonene were statistically higher at nail salons with more than 30 daily customers (median: 6.18 ppb) than at nail salons with less than 30 daily customers (median: 2.30 ppb). Hence, nail salons with a large number of daily customers might frequently use cleaning and disinfection products containing *d*-limonene to clean the tables after nail services. Elevated levels of *d*-limonene have also been associated with cleaning practices in occupational settings [41] and the use of personal care products or cleaning agents indoors. However, the elevated levels of *d*-limonene may not directly result in NSTs’ exposure to *d*-limonene due to uncertainty of the cleaning time (during or after work hours) and types of cleaning product used that were not collected in this study.

In addition to VOCs, we tested the feasibility of measuring airborne phthalates using SWBs. While SWBs have been recently used as a passive dosimeter to characterize SVOCs among firefighters, farmers, and general populations [28,29,30,31,32], only two pilot studies, to our knowledge, measured several phthalates using SWBs. One study was conducted for exposure assessment for nine Vietnamese NSTs in Boston, MA [26]. Another study examined personal exposure to phthalates and organic phosphate esters among 49 NSTs in Toronto, Ontario, Canada [42]. Although the validation of SWBs on phthalates has not been examined in this study, recent studies validated the use of SWBs collecting phthalates in nail salons and general environments. For example, Nguyen et al. (2022) reported the recoveries of matrix spikes were between 70% and 109% for 10 phthalates among NSTs in Toronto, Ontario [42]. Hong et al. (2021) also used modified SWBs to estimate personal exposure to phthalates and other organic chemicals. The authors reported that the extraction efficiency was greater than 99% and the recovery of DiBP and DEHP were equally 94% in field blanks and 108% and 116% in environmental samples, respectively [43].

In this pilot study, we detected six phthalates from all 10 nail salons and the concentrations of DEHP (n = 10, median: 392 ppb) were highest. DEHP is a plasticizer that makes nail polish softer, more flexible, and more durable. Young et al. (2018) found that DEHP was detected in 39 out of the 40 nail polishes available in the U.S. market [44]. Moreover, Tran and Kannan (2015) observed that levels of DEHP in hair and nail salons (n = 6) was 2–10-fold higher than in residential homes, offices, and schools [27]. Biomarker studies confirmed that NSTs had significantly higher levels of phthalate metabolites (including DEHP) than the U.S. adult population as per the National Health and Nutrition Examination Survey (NHANES) [45]. Varshavsky et al. (2020) also reported that urinary monoethylhexyl phthalate (MEHP), a metabolite of DEHP, among Vietnamese female NSTs (n = 15, GM = 3.5 µg/L) was significantly higher than in those of female Asian Americans from 2011–2012 NHANES data (n = 97, GM = 1.6 µg/L) [46]. Exposure to DEHP, an endocrine-disrupting chemical, may cause adverse reproductive and developmental effects [47,48,49]. Unlike VOCs, DEHP and other phthalates with relatively low volatility may linger longer in the air and persist in indoor environments during non-business hours and even for several years [50].

While 31 VOCs and six phthalates were commonly measured in nail salons in NJ and NY, the large variability of measured VOCs and phthalates at nail salons may be associated with multiple factors such as state and local regulations, nail salon characteristics, and other environmental factors. For instance, the median concentration of toluene in NJ (9.85 ppb) was approximately 2-fold higher than in NY (5.17 ppb). Toluene has been widely measured to evaluate the indoor air quality of nail salons since toluene is one of the primary ingredients found in nail products [8,51,52]. The higher levels of toluene in NJ nail salons might be related to the larger number of average daily customers visiting NJ nail salons (40 ± 19 customers) than NY nail salons (33 ± 23 customers), although there was no statistically significant difference in toluene levels based on average daily customers. An intervention study conducted at Vietnamese nail salons in California reported that nail salons that served 20 or more daily customers showed significantly higher toluene concentrations (GM: 60 ppb) than nail salons that served less than 20 daily customers (GM: 40 ppb) [51]. In our study, we found that toluene concentrations were significantly higher (approximately 3 fold) at nail salons with 15 or more manicure and pedicure tables than at those with less than 15 tables (we used the number of tables as a proxy for the number of nail services provided in the salons). As described above, nail salons serving more customers showed significantly higher levels of *d*-limonene than nail salons with fewer customers.

Similar to existing pilot studies on nail salons, there are several limitations to this pilot study as well. The sample size (n = 20) was small and there was selection bias due to convenience sampling (although we worked with local nail salon organizations in NJ and NY as community partners). As described earlier, passive samplers for VOCs and phthalates were not co-located with active samplers to determine the accuracy of collecting these organic chemicals. Indoor air temperature and airflow inside nail salons were not measured during the sampling. However, sampling errors due to temperature variation in nail salons (+/−5 °C from 25 °C) are likely less than 1 percent [53]. Sampling rates (oversampling) are likely increased at the higher velocity for certain types of passive samplers (e.g., palms tube or DuPont sampler), although the 3M OVM used in this study may not oversample since no oversampling was observed when face velocities were up to 2 m/s [53]. Thus, we do not expect significant sampling errors for VOC sampling. As sampling campaigns were conducted during the COVID-19 pandemic, the results might not be representative of “normal” nail salon services. Due to various logistical reasons, including COVID-19 safety issues, we were not able to conduct a walk-through survey to validate whether the ventilation systems were on or off during the provision of nail services. While we observed that NY nail salons used more organic nail products than NJ nail salons, we did not validate the types of nail products or examine the material safety data sheet in this study. Despite these limitations, this study showed the feasibility of partnering with local nail salon organizations in NJ and NY to recruit 20 nail salons within three months despite the COVID-19 pandemic. We also measured elevated concentrations of *d*-limonene, a possible indication of the increased use of cleaning and disinfection agents during the pandemic. Comprehensive measurements of VOCs and phthalates in the air were achieved using traditional (OVM badge) and innovative SWB passive samplers. Finally, we provided individual results to nail salon owners and answered questions regarding the results to help them make informed decisions on the measures to be taken to reduce VOC and phthalates in their salons.

## 5. Conclusions

The results of this pilot study suggest that Korean and Chinese NSTs are exposed to various levels of VOCs and phthalates from nail and disinfection products at their workplaces during COVID-19. Toluene, d-limonene, and DEHP were highest compounds in NJ and NY nail salons. Some VOCs (i.e., MMA, methylene chloride, and α-pinene) but not all VOCs and phthalates were different between NJ and NY salons. Large numbers of customers and the provision of acrylic services at the nail salons were observed to lead to elevated exposure to these chemicals. Future studies should assess personal exposure to a mixture of VOCs, phthalates, and other hazardous compounds for NSTs. Additionally, the types of nail products and cleaning products used at nail salons should be examined to understand the potential impact of nail products on health. Accurate information on ventilation and its proper use must also be assessed. Future studies must work with community organizations to mitigate potential exposure to nail products among Asian immigrant NSTs.

## Figures and Tables

**Table 1:** Nail salon characteristics and environment (N = 20).

	New Jersey (n = 10)		New York (n = 10)		Total (N = 20)	
	M (SD)	N (%)	M (SD)	N (%)	M (SD)	N (%)
Number of employees	6.80 (2.62)		8.30 (4.50)		7.55 (3.66)	
Daily customers during data collection	40.00 (19.15)		32.50 (23.01)		36.25 (20.96)	
Daily open hours during data collection	9.56 (0.50)		9.61 (1.12)		9.59 (0.84)	
Number of manicure tables	7.70 (2.21)		7.50 (1.78)		7.60 (1.96)	
Number of pedicure tables	6.90 (2.42)		6.70 (2.00)		6.80 (2.17)	
Doors						
1		2 (20%)		4 (40%)		6 (30%)
2 or more		8 (80%)		6 (60%)		14 (70%)
Windows						
0		7 (70%)		7 (70%)		14 (70%)
1 or more		3 (30%)		3 (30%)		6 (30%)
Has a ventilation system		6 (60%)		7 (70%)		13 (65%)
Ventilated manicure station		4 (40%)		3 (30%)		7 (70%)
Ventilated pedicure station		1 (10%)		1 (10%)		2 (20%)
Table fan connected to duct to vent contaminated air outside		2 (20%)		1 (10%)		3 (30%)
Exhaust hood to vent contaminated airoutside		3 (30%)		4 (40%)		7 (70%)
Use of products with all natural or organic ingredients		4 (40%)		8 (80%)		12 (60%)
Type of services						
Regular manicure/pedicure		10 (100%)		10 (100%)		20 (100%)
Artificial nail (UV gels)		10 (100%)		10 (100%)		20 (100%)
Artificial nail (Acrylics)		1 (10%)		5 (50%)		6 (30%)
Artificial nail (Dip powders)		8 (80%)		10 (100%)		18 (90%)
Spa/Facial/Waxing		8 (80%)		6 (60%)		14 (70%)
Eyelash extension		3 (30%)		4 (40%)		7 (35%)

**Table 2:** Summary of descriptive statistics for 31 VOCs and 7 phthalates measured at 20 nail salons in NJ and NY from February to May 2021.

Class	Chemicals	LOD ^a^	New Jersey			New York			Total		
			N > LOD	Mean ± SD	Median	N > LOD	Mean ± SD	Median	N > LOD	Mean ± SD	Median
VOCs	Butadiene	0.12	1	N/A	<0.12	0	N/A	<0.12	1	N/A	<0.12
	Pentane	0.11	10	1.86 ± 1.50	1.55	10	2.36 ± 3.60	1.22	20	2.11 ± 2.70	1.36
	Isoprene	0.09	4	N/A	<0.09	4	N/A	<0.09	8	N/A	<0.09
	Methylene Chloride	0.01	10	0.63 ± 0.43	0.70	10	1.65 ± 1.74	1.14	20	1.14 ± 1.34	1.03
	n-Hexane	0.11	10	0.21 ± 0.09	0.18	8	0.89 ± 1.60	0.19	18	0.55 ± 1.16	0.18
	Methyl Cyclopentane	0.03	3	N/A	<0.03	3	N/A	<0.03	6	N/A	<0.03
	Methyl Ethyl Ketone	0.13	1	N/A	<0.37	0	N/A	<0.37	1	N/A	<0.37
	Chloroform	0.07	8	0.45 ± 0.32	0.50	5	1.31 ± 3.33	0.11	13	0.88 ± 2.34	0.37
	2,3-Dimethyl Pentane	0.07	2	N/A	<0.07	5	1.70 ± 3.27	0.08	7	N/A	<0.07
	Trichloroethylene	0.07	0	N/A	<0.07	3	N/A	<0.07	3	N/A	<0.07
	Carbon Tetrachloride	0.03	5	0.04 ± 0.02	0.03	2	N/A	<0.03	7	N/A	<0.03
	Benzene	0.21	10	0.58 ± 0.26	0.54	10	1.06 ± 1.55	0.43	20	0.82 ± 1.11	0.51
	Methyl Methacrylate	0.09	7	21.74 ± 62.27	1.77	3	N/A	<0.09	10	14.44 ± 46.13	0.12
	Toluene	0.03	10	12.74 ± 9.96	9.85	10	8.39 ± 9.30	5.17	20	10.57 ± 9.64	7.03
	Ethyl Methacrylate	0.08	7	9.85 ± 30.46	0.12	6	3.08 ± 7.77	0.55	13	0.25 ± 0.22	0.21
	Tetrachloroethylene	0.06	10	0.35 ± 0.71	0.13	10	2.35 ± 4.67	0.14	20	1.35 ± 3.41	0.13
	Ethyl Benzene	0.03	10	0.28 ± 0.12	0.23	10	0.22 ± 0.30	0.11	20	0.25 ± 0.22	0.21
	Nonane	0.05	2	N/A	<0.05	5	0.10 ± 0.14	0.04	7	N/A	<0.05
	m,p-Xylenes	0.10	10	0.73 ± 0.34	0.77	10	0.70 ± 0.85	0.36	20	0.71 ± 0.63	0.62
	o-Xylene	0.10	10	0.25 ± 0.13	0.26	10	0.23 ± 0.27	0.11	20	0.24 ± 0.21	0.19
	Styrene	0.02	8	0.10 ± 0.08	0.09	6	0.10 ± 0.22	0.03	14	0.10 ± 0.16	0.04
	α-Pinene	0.09	10	0.81 ± 0.87	0.47	10	0.54 ± 0.88	0.22	20	0.67 ± 0.86	0.33
	n-Decane	0.04	3	N/A	<0.04	4	N/A	<0.04	7	N/A	<0.04
	1,3,5-Trimethylbenzene	0.03	2	N/A	<0.03	3	N/A	<0.03	5	N/A	<0.03
	1-Ethyl-2-methylbenzene	0.02	3	N/A	<0.02	4	N/A	<0.02	7	N/A	<0.02
	β-Pinene	0.09	10	0.81 ± 1.51	0.32	9	0.84 ± 1.59	0.31	19	0.82 ± 1.51	0.32
	1,2,4-Trimethylbenzene	0.02	2	N/A	<0.02	6	0.03 ± 0.03	0.02	8	N/A	<0.02
	d-Limonene	0.10	10	7.77 ± 8.14	6.14	10	6.50 ± 7.58	2.52	20	7.14 ± 7.68	4.21
	1,2,3-Trimethylbenzene	0.03	9	0.10 ± 0.06	0.11	10	0.13 ± 0.12	0.09	19	0.11 ± 0.10	0.10
	1,4-Dichlorobenzene	0.05	2	N/A	< 0.05	1	N/A	<0.05	3	N/A	<0.05
Phthalates	Di(2-Ethylhexyl) Phthalate (DEHP)	2	3	445.7 ± 137.2	469.72	7	394.3 ± 72.63	390.92	10	409.7 ± 91.20	391.80
	Di-n-octyl Phthalate (DnOP)	2	3	10.83 ± 4.11	8.46	7	25.96 ± 5.75	25.82	10	21.42 ± 8.90	23.49
	Diisobutyl Phthalate (DiBP)	2	3	106.4 ± 11.53	102.1	7	120.2 ± 44.87	97.15	10	115.5 ± 37.80	99.61
	Diethyl Phthalate (DEP)	2	3	200.8 ± 137.7	142.0	7	226.0 ± 225.7	166.9	10	218.4 ± 195.8	154.4
	Di-n-butyl Phthalate (DnBP)	2	3	61.95 ± 37.31	53.86	7	67.13 ± 25.71	67.07	10	65.58 ± 27.50	61.28
	Benzylbutyl Phthalate (BzBP)	2	3	7.72 ± 0.68	7.50	7	25.26 ± 25.96	17.58	10	20.00 ± 22.83	12.04
	Dimethy Phthalate (DMP)	10	0	N/A	<10	0	N/A	<10	0	N/A	<10

^a^ LODs for VOCs were estimated based on average sampling duration (4771 min) from 20 nail salons at 25 °C under 1 atmospheric pressure. The units for VOCs and phthalates are ppb and ng/g-wristband, respectively.

## Data Availability

The data presented in this study are available on request from the corresponding author. The data are not publicly available due to proprietary data (nail salon).

## Abbreviations

**Class**	
MC	Methylene Chloride
B	Benzene
MMA	Methyl methacrylate
T	Toluene
EMA	Ethyl methacrylate
TCE	Tetrachloroethylene
E	Ethyl benzene
X	m,p-Xylenes
X	o-Xylene
**Phthalates**	
DEHP	Di(2-Ethylhexyl) Phthalate
DnOP	Di-n-octyl phthalate
DiBP	Diisobutyl phthalate
DEP	Diethyl phthalate
DnBP	Di-n-butyl phthalate
BzBP	Benzylbutyl phthalate
DMP	Dimethy phthalate
